# Keynote 1

**DOI:** 10.1093/eurpub/ckac092.001

**Published:** 2022-08-29

## Abstract

**Title of HEPA 2022 Keynote presentation:**

Leveraging Citizen Science as part of an Ecosystem Approach to Health-Enhancing Physical Activity.

**Description of HEPA 2022 Keynote presentation:**

During the past 50 years, the positive effects of health-enhancing physical activity have continued to expand exponentially; yet many populations worldwide remain insufficiently active and, thus, unable to fully realize the benefits that regular physical activity offers. While a substantial number of evidence-based physical activity interventions have been successfully tested, few have specifically targeted the local environmental contexts that have a key impact on daily physical activity levels across the life course. A major focus of this presentation is to describe a technology-enabled participatory research model, called the Our Voice global citizen science research initiative, which actively engages community residents worldwide in assessing, analyzing, and building practical solutions to local environmental barriers to health-enhancing physical activity. Current results of Our Voice projects, which span six continents and have included residents ages 9 to >95, indicate that community residents themselves, irrespective of circumstances, age, or culture, may in fact be the planet’s most valuable “renewable resource” for helping to change local environments for active and healthy living.

